# Inflammatory Signalling in Fetal Membranes: Increased Expression Levels of TLR 1 in the Presence of Preterm Histological Chorioamnionitis

**DOI:** 10.1371/journal.pone.0124298

**Published:** 2015-05-12

**Authors:** Gareth J. Waring, Stephen C. Robson, Judith N. Bulmer, Alison J. Tyson-Capper

**Affiliations:** 1 Directorate of Women’s services, Newcastle Upon Tyne NHS Foundation Trust, Newcastle Upon Tyne, United Kingdom; 2 Institute of Cellular Medicine, Newcastle University, Newcastle Upon Tyne, United Kingdom; University of Cambridge, UNITED KINGDOM

## Abstract

Histological chorioamnionitis (HCA) is an established marker of ascending infection, a major cause of preterm birth. No studies have characterised the global change in expression of genes involved in the toll-like receptor (TLR) signalling pathways in the presence of HCA in the setting of preterm birth (pHCA). Fetal membranes were collected immediately after delivery and underwent histological staging for inflammation to derive 3 groups; term spontaneous labour without HCA (n = 9), preterm birth <34 weeks gestation without HCA (n = 8) and pHCA <34 weeks (n = 12). Profiling arrays ran in triplicate for each group were used to determine the expression of 84 genes associated with TLR signalling and screen for genes of interest (fold change >2; p<0.1). Expression of identified genes was validated individually for all samples, relative to GAPDH, using RT-PCR. Expression of TLR 1, TLR 2, lymphocyte antigen 96, interleukin 8 and Interleukin-1 receptor-associated kinase-like 2 was increased in pHCA (p<0.05). Degree of expression was positively associated with histological staging of both maternal and fetal inflammation (p<0.05). The inflammatory expression profile at the maternal/fetal interface associated with pHCA, a reflection of ascending infection, is extremely heterogeneous suggesting polymicrobial involvement with activation of a common pathway. Antagonism of TLR 1 and TLR 2 signalling in this setting warrants further assessment.

## Introduction

Preterm birth (PTB), birth less than 37 weeks completed gestation, is a major cause of perinatal morbidity and mortality. In the developed world it accounts for 75% of perinatal mortality and >50% long term morbidity [1]. Of the three precursor groups of PTB, spontaneous preterm labour with intact membranes and preterm premature rupture of the membranes (pPROM) are considered spontaneous preterm birth (sPTB). sPTB account for 65–75% of all PTB [1].

Infection is the principle pathologic process with an established causal link to sPTB and a defined molecular pathophysiology. Ascending infection via the vagina and cervix is the most common pathway [2–4]. Histologic chorioamnionitis (HCA) is the most specific and sensitive marker for infection [5–8] and correlates with periventricular leucomalacia (RR 1.9; 95% CI, 1.4–2.5) and cerebral palsy (RR 1.6; 95% CI, 0.9–2.7)[9]. It has been suggested that there is some benefit to HCA in that it is associated with a reduction in the incidence and severity of respiratory distress syndrome, although this is accompanied by an increased risk of bronchopulmonay dysplasia [10].

Chorioamnionitis refers to the presence of acute inflammation in the fetal membranes (chorion and amnion). There is strong relationship between HCA and gestational age at delivery; HCA is identified in 10% of term deliveries, 30% of preterm labour with intact membranes and 50% of pPROM with a highest finding of 80–90% in miscarriages between 20 and 24 weeks [11]. A major drawback in developing new interventions to treat sPTB is our poor understanding of the physiology of human parturition at term. Parturition involves a common pathway which manifests as uterine contractions, cervical ripening and decidual activation, culminating in membrane rupture and birth. This has been observed in both term and preterm birth and whilst the mechanism is not fully understood, the evidence supports the role of inflammatory mediators [12].

The innate immune system is the first line of defence against invading microorganisms at the interface of the maternal and fetal compartments. The system is responsible for establishing and maintaining a suitable microenvironment for pregnancy, recognising ‘infectious non-self’ (microorganisms) and ‘non infectious self’ (mother, placenta, fetus). Microorganisms are identified by pattern recognition receptors such as Toll-like receptors (TLRs) which recognise pathogen associated molecular patterns (PAMPs) unique to the microorganisms. Ligation of TLRs by PAMPs results in an inflammatory response generated against the invading pathogen. Upon ligand recognition TLRs recruit MyD88, an intracellular signalling adaptor protein, leading to a kinase cascade which triggers activation of the nuclear factor-kappa B (NF-кB) signalling pathway. This leads to a rapid change in gene expression producing chemokines, cytokines and antimicrobial peptides [13]. TLR-3 and TLR-4 are also able to signal in a MyD88 independent pathway (TRIF) to trigger an antiviral response [14]. There are 11 known mammalian TLRs (1–11). Expression of TLRs has been investigated in gestational tissue; mRNA expression of TLRs 1–10 and accessory proteins (e.g. CD14) has been described in the placenta [15] and temporal expression changes in TLR-2 and TLR-4 have been reported in term and preterm myometrium [16]. We have shown that TLR-4 and several cofactors of TLR activation (e.g. CD-14, MyD88 and MD-2) are up-regulated in the lower region of the uterus in advancing pregnancy but not the upper region [17]. Less is known about TLR expression and activation in the fetal membranes. Chorioamnionitis at both term and preterm is associated with increased protein expression of TLR-2 and TLR-4. TLR-2 expression has been shown to be restricted to the basal surface of the amniotic epithelial membrane in preterm labour without HCA but diffusely present across the entire epithelial cytoplasm with HCA [18]. TLR-4 has also been shown to translocate from the apical to the basal membrane in the presence of HCA [19]. Studies have traditionally focused on individual inflammatory mediators but with the advent of qPCR array technology it is now possible to examine global changes of many genes simultaneously in a single experiment. This approach has been used to assess the transcriptome of fetal membranes at term [20] and the cytokine expression at term and preterm in fetal membranes has also been reported [21].

In this study we aimed to describe the changes in gene expression in the TLR signalling pathway associated with preterm chorioamnionitis. We used qPCR array technology to screen 84 genes in the signalling pathway and sought to confirm changes in individual genes using RT-PCR.

## Materials and Methods

### Study design: tissue selection

A prospective study was designed to examine the differential gene expression in both amnion and chorion with and without preterm HCA. Women admitted in spontaneous labour were recruited and categorised into 3 different groups; term spontaneous labour without chorioamnionitis (TSL^-CA^), preterm spontaneous labour without chorioamnionitis (PTL^-CA^), preterm spontaneous labour with chorioamnionitis (PTL^+CA^). Over a 12 month period, 29 women were consented and recruited into this study. All participants were recruited from the Newcastle Upon Tyne NHS Foundation Trust Hospitals. After histological phenotyping the groups were made up as follows; TSL^-CA^ (n = 9), PTL^-CA^ (n = 8), PTL^+CA^ (n = 12).

Approval for the study was granted by Newcastle and North Tyneside 1 Research Ethics Committee (Ref:10/H0906/71) and Newcastle Upon Tyne NHS Foundation Trust. Written consent was taken from all participants to collect and store the samples and to use medical outcome data from the patient and neonate for the purpose of the study. Labour was defined as the presence of regular spontaneous uterine contractions accompanied by progressive cervical dilation that lead to delivery. Term was defined as a gestational age of ≥37 weeks. Samples were only collected from women delivering <34 weeks’ gestation to capture early preterm birth.

### Tissue preparation

Chorioamniotic membranes and placenta were collected immediately after delivery from participants. Pieces of chorioamniotic membranes were sterile dissected from the placental edge and placed in sterile ice cold phosphate buffered saline solution (1x PBS). Chorioamniotic membranes were washed thoroughly in 1xPBS to remove blood and debris. 4–5cm pieces of membrane were dissected out, amnion was separated from chorion and decidua parietalis was scraped off the chorion. Amnion and chorion were stored at -70°C. For histological examination of term control placentas, sections of the amniochorioinic membranes, umbilical cord and chorionic plate were fixed in 10% neutral buffered formalin, embedded in paraffin wax, sectioned at 3μm and stained with haematoxylin and eosin (H&E) for histological assessment. Preterm placentas were sent for routine histopathological assessment. Two membrane rolls (amnion and chorion laeve intact), two cross sections of umbilical cord and two full thickness sections of placental parenchyma including maternal and fetal surfaces were assessed for the presence of maternal and fetal inflammatory responses by a consultant gynaecological/placental histopathologist (JNB) using standard criteria [22]. Maternal inflammatory responses were assessed by the presence of chorionitis and subchorionitis (stage 1 maternal inflammatory response) and chorioamnionitis (stage 2 maternal inflammatory responses). Fetal inflammatory responses were assessed in chorionic arteries and umbilical cord vessels. Samples were designated as showing HCA with a maternal inflammatory response of stage 2 or above.

### RNA isolation and cDNA synthesis

Total RNA was extracted using QIAzol/TRIzol (Qiagen) in accordance with the manufacturer’s protocol. After the ethanol precipitation step RNA was further cleaned using the Qiagen RNeasy mini kit. This included an on-column DNase I treatment step. Integrity, quantity and purity of the RNA was verified by the A260:A230 ratio, A260:A280 ratio and gel electrophoresis. Single stranded cDNA was synthesised using Qiagen RT^2^ First Strand kit in accordance with the manufacturer’s protocol.

### qPCR

PCR array for TLR pathway was performed by using RT^2^-Profiler PCR array platform (Toll-like Receptor signalling pathway array PAHS-018, SABiosciences, Qiagen). A 96-well plate contains gene-specific primer sets for 84 relevant genes for TLR signalling pathway, 5 housekeeping genes and 2 negative controls. An experimental cocktail of diluted first strand cDNA, nuclease free water and RT^2^ qPCR master mix (Sybr Green) was mixed in accordance with the manufacturer’s protocol. Arrays were performed using ABI StepOnePlus thermocycler. For each of the experimental groups, qPCR arrays were performed in triplicate for both amnion (n = 9) and chorion (n = 9). Genes showing significant change in expression on signalling arrays were validated individually using qPCR. Real-time PCR was performed on cDNA using inventoried TaqMan (Applied Biosystems) and TaqMan Universal Master Mix II(Applied Biosystems). TaqMan GAPDH assay was selected as an endogenous control due to its consistent results as a house keeping gene in the signalling array phase of the study. Each assay was performed in triplicate (3 biological replicates per plate) for both amnion (n = 29) and chorion (n = 29) for each sample.

### Statistical analyses

Clinical data was analysed using GraphPad Prism 5 software. Comparison of means was by unpaired t-test with categorical data analysed with Fisher’s exact test. Analysis of both the signalling arrays and the individual gene qPCR was carried out using Sabiosciences PCR array data analysis web portal[23]. To determine genes of interest, volcano plots were used comparing each of the three groups to each other for both amnion and chorion. Students t test was used to test for differential expression when comparing one group to another. A false discovery rate (p) threshold of 0.1 in conjunction with a fold change threshold of 2 to assign gene significance in the array analysis stage[24]. This is to screen the large amount of data generated and hone in on genes of interest. The same test and analysis software was used for the qPCR validation but here we used a threshold of p<0.05 to infer statistical significance. Correlation between gestational age and gene expression was estimated by least squares linear regression modelling using a significance of 0.05. Correlation between staging of inflammation (maternal and fetal) and gene expression was also estimated using least squares linear regression modelling; a p< 0.05 was used to define statistical significance.

## Results

Maternal characteristics and fetal outcome data was collected and can be seen in Tables 1 and 2. The characteristics of both preterm birth groups and the term group were very similar and did not differ significantly with the expected exceptions of birth weight, gestational age at delivery and percentage receiving antenatal corticosteroids. Fetal outcomes are displayed in Table 3. A composite measure of immediate problems at birth was similar between both the preterm birth groups. A higher rate of bronchopulmonary dysplasia was found in the PTL^+CA^ group however given the numbers this was not statistically significant. There was a single neonatal death, this occurred in the PTL^+CA^ group.

**Table 1:** Maternal characteristics.

	TSL^-CA^	p*	PTL^-CA^	p**	PTL^+CA^	p***
Mean materal age (yrs)	30.9	0.0634	25.9	0.4010	28.6	0.4658
Mean BMI	22.84	0.6064	23.88	0.9567	24.03	0.5016
Mean birthweight (g)	3211.1	*0*.*0001*	1963.5	*0*.*0289*	1404.9	*0*.*0001*
Mean gestational age (weeks + days)	40+2	*0*.*0001*	30+5	0.5377	29+5	*0*.*0001*
% Smoker	11	0.29	38	1.00	27	0.36
% Previous Preterm birth	0	0.2059	25	0.6557	38	0.0537
% Antenatal Corticosteroids	0	*0*.*0004*	88	1.0000	92	*0*.*0001*

Difference in means assessed using unpaired t-test (p<0.05). Categorical data assessed using Fishers exact test (p<0.05).

*Comparison between TSL^-CA^ and PTL^-CA^

** Comparison between PTL^-CA^ and PTL^+CA^

*** Comparison between TSL^-CA^ and PTL^+CA^

**Table 2:** Fetal outcome data.

	PTL^-CA^ n(%)	PTL^+CA^ n(%)	p
Early fetal complications	5 (63)	8 (67)	1.0000
Bronchopulmonary dysplasia	1 (13)	4(33)	0.6027
Neonatal death	0 (0)	1 (8)	1.0000

Data on preterm infants assessed using fishers exact test (p<0.05).

**Table 3:** Gene expression: PTL^+CA^ vs TSL^-CA^.

Gene	Amnion	p	Chorion	p
**HMGB1**	-1.2303	0.763159	1.1146	0.695966
**IL8**	319.8713	***0*.*024156***	54.6698	***0*.*037295***
**IRAK2**	109.7893	***0*.*008412***	27.9439	***0*.*029515***
**LY96**	105.4454	***0*.*024194***	25.9928	***0*.*000296***
**MyD88**	2.9342	***0*.*040499***	1.0541	0.708289
**SARM1**	4.1177	***0*.*010122***	1.025	0.455544
**SIGIRR**	3.3354	0.110863	2.142	0.722095
**TIRAP**	1.901	0.121438	1.0724	0.267937
**TLR1**	82.5792	***0*.*035394***	18.8113	***0*.*001473***
**TLR2**	80.3276	***0*.*045811***	9.3475	***0*.*000451***
**TLR4**	38.0362	0.0512	1.1705	0.275484
**TLR6**	2.3639	0.034343	1.6584	0.277442

Mean expression values shown. Students t-test used to test for significance (p<0.05). Expression normalised to GapDH. Gene expression assessed by fold change (2^ΔΔCT^).

### TLR profiling array signalling in chorioamnionitis

**Fig 1:**
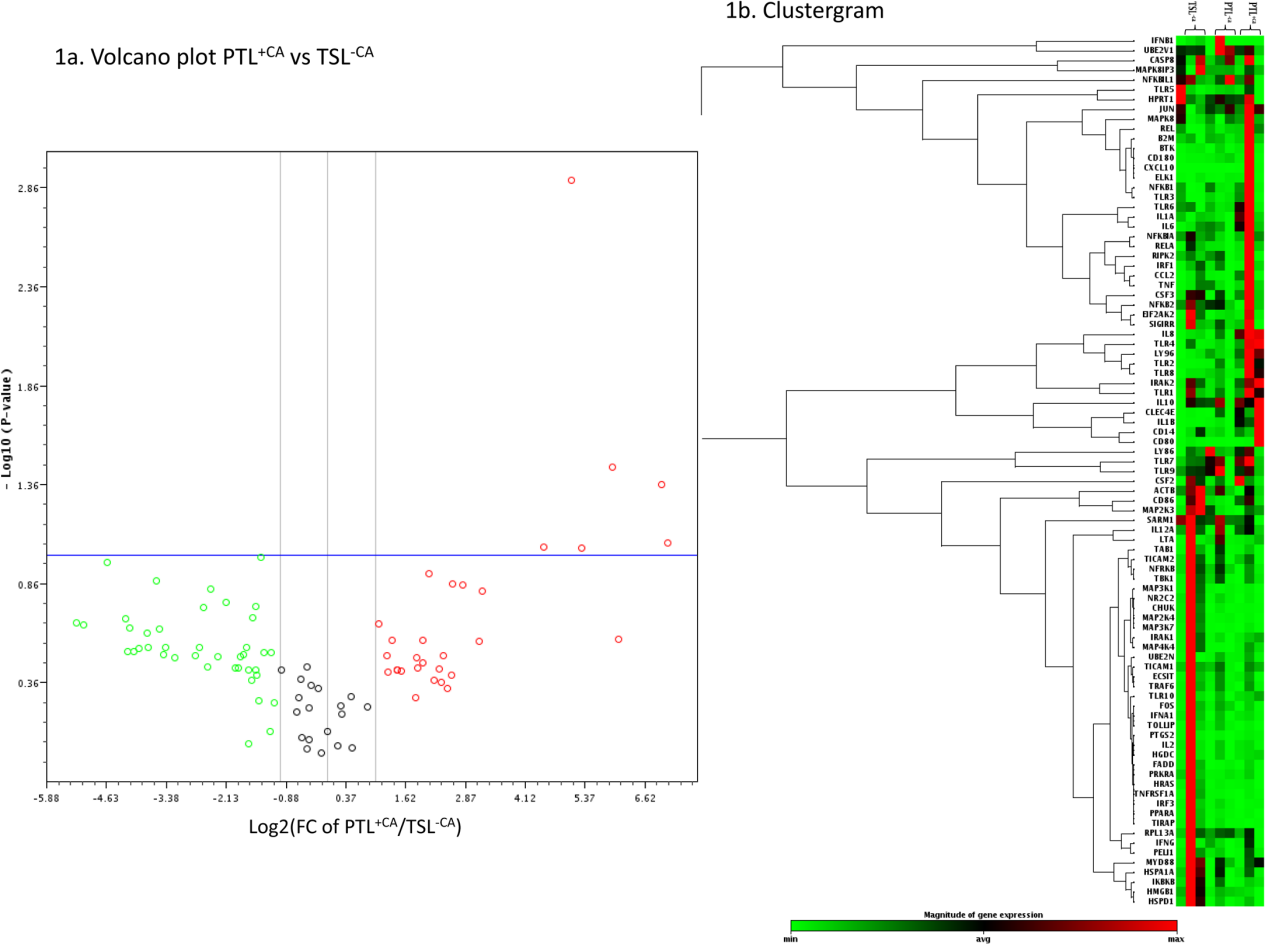
Screening for genes interest: Analysis of TLR pathway signalling array. Volcano plots were used to identify genes showing significant expression. The example shown here is a volcano plot of PTL^+CA^ vs TSL^-CA^ in the amnion (2a). The plot shows the values for all genes in the qPCR array plotted against fold changes. On the Y-axis, values higher than the blue line threshold represent significant genes with an adjusted probability value of <0.1. On the X-axis, values outside the grey lines represent a fold change of either ≥2 or ≤-2. The clustergram with heat map (2b) shows all 84 genes in qPCR array for each group in the amnion, this was used to visually identify the pattern of which clusters of genes showed higher expression in each group.

Fig 1 shows an example of the data generated from the array analysis for amnion from PTL^+CA^ and PTL^-CA^. In the amnion; comparing PTL^+CA^ and PTL^-CA^ there were 4 genes showing increased expression, while comparing PTL^+CA^ and TSL^-CA^ 7 genes showed a change in expression and comparing PTL^+CA^ and TSL^-CA^ there was 1 gene that changed (fold change +/- 2; p<0.1). In the chorion; comparing PTL^+CA^ and PTL^-CA^ there were 3 genes showing a change in expression, when comparing PTL^+CA^ and TSL^-CA^ 4 genes showed changes and comparing PTL^+CA^ and TSL^-CA^ there were 3 gene showing changes. The full signalling array analysis can be found. (S1, S2 and S3 Tables)

Overall the expression profile, changed for 13 different genes in the presence or absence of HCA. These were designated genes of interest; TLR1, TLR2, TLR4, TLR6, TLR7, IL-8, HMGB1, SIGIRR, MyD88, IRAK2, LY96, SARM1 and TIRAP. To validate the array work expression of each of these individual genes was assessed for all samples in the study. In this validation we did not detect TLR 7 expression and therefore data for TLR 7 is not shown.

### Expression profiling in the absence of inflammation

All samples from all three groups in the study were analysed. Firstly we compared PTL^-CA^ and TSL^-CA^. In the absence of HCA there was increased expression of TLR 1 (32.7 fold increase; p = 0.002), TLR 2 (12.0 fold increase; p = 0.02) and TLR 4 (18.4 fold increase; p = 0.008) in the amnion of PTL^-CA^ (S4 Table). This observation was not replicated in the chorion, although there was a trend for higher expression in the PTL^-CA^ group. LY96 (41.1 fold increase; p = 0.002), MyD88 (2.5 fold increase; p = 0.009), IRAK2 (13.7 fold increase; p = 0.006) and SARM1 (8.8 fold increase; p = 0.003) all showed increased expression in the amnion of PTL^-CA^.

**Fig 2:**
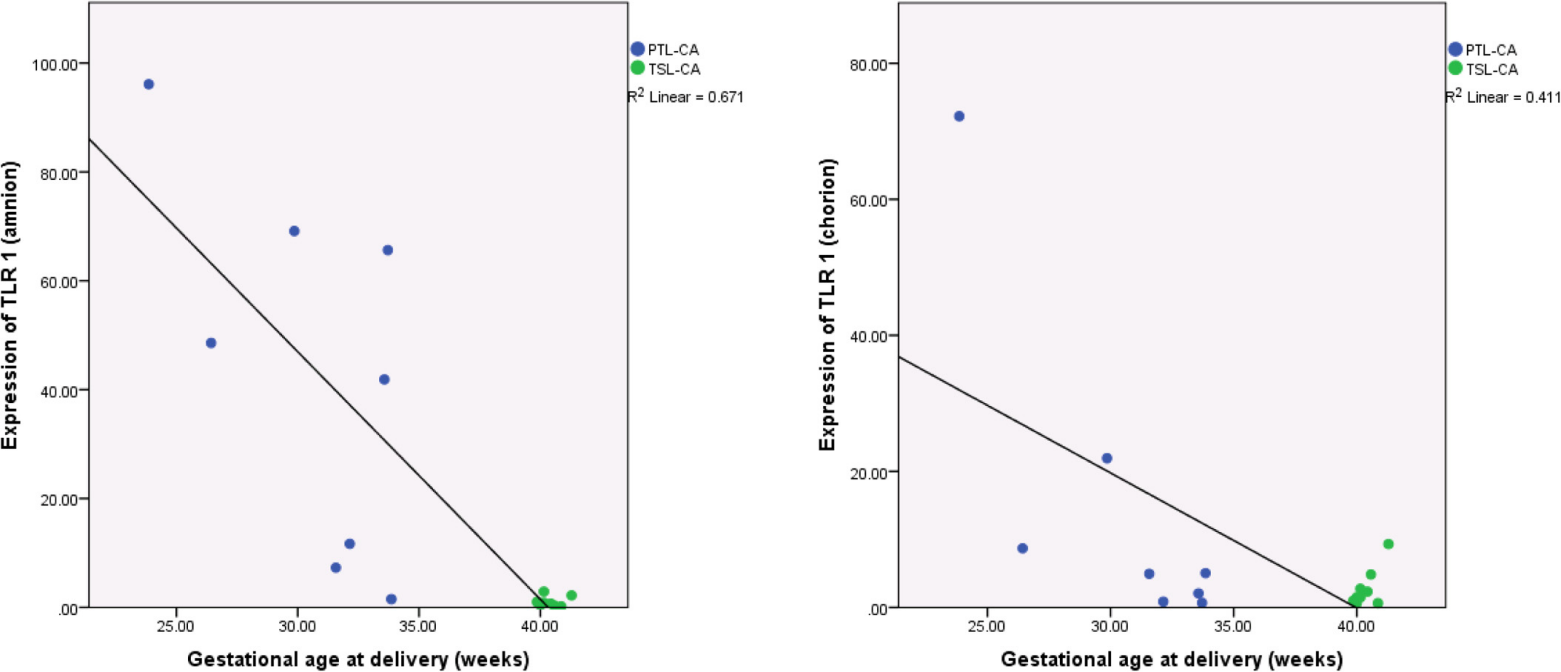
Expression of TLR 1 is negatively related to gestational age. Least squares linear regression is used to assess correlation between expression of TLR 1 and gestational age in the absence of chorioamnionitis (PTL^-CA^ and TSL^-CA^). All individual samples with histological confirmation of the absence of CA are shown. TLR 1 shows a strong correlation between decreasing gestational age and increased expression (R^2^ = 0.671, p<0.001). The correlation is also present in the chorion (R^2^ = 0.411, p = 0.006). Expression normalised to GapDH. Gene expression assessed by fold change (2^ΔΔCT^).

In both amnion and chorion lower gestational age was significantly correlated with increased expression of TLR 1 (Fig 2), LY96, IRAK2 and the negative regulator SIGIRR. TLR 4, SARM1, MyD88 and TIRAP showed a significant correlation of increased expression with lower gestational age in the amnion only. TLR 2, TLR 6 and IL8 showed the same pattern though just in the chorion (S5 Table).

### The effect of chorioamnionitis on gene expression

To assess the effect of HCA, gene expression from PTL^+CA^ was compared firstly to TSL^-CA^. In keeping with our findings comparing PTL^-CA^ and TSL^-CA^, MyD88 and SARM1 showed increased expression in the amnion, suggesting that the increased expression of these genes is a feature of the membrane gestational age rather than inflammatory status. TLR 1 and 2 showed increased expression in both chorion and amnion (Table 3). IL8, IRAK2 and LY96 mirrored this pattern with increased expression in both tissues.

**Fig 3:**
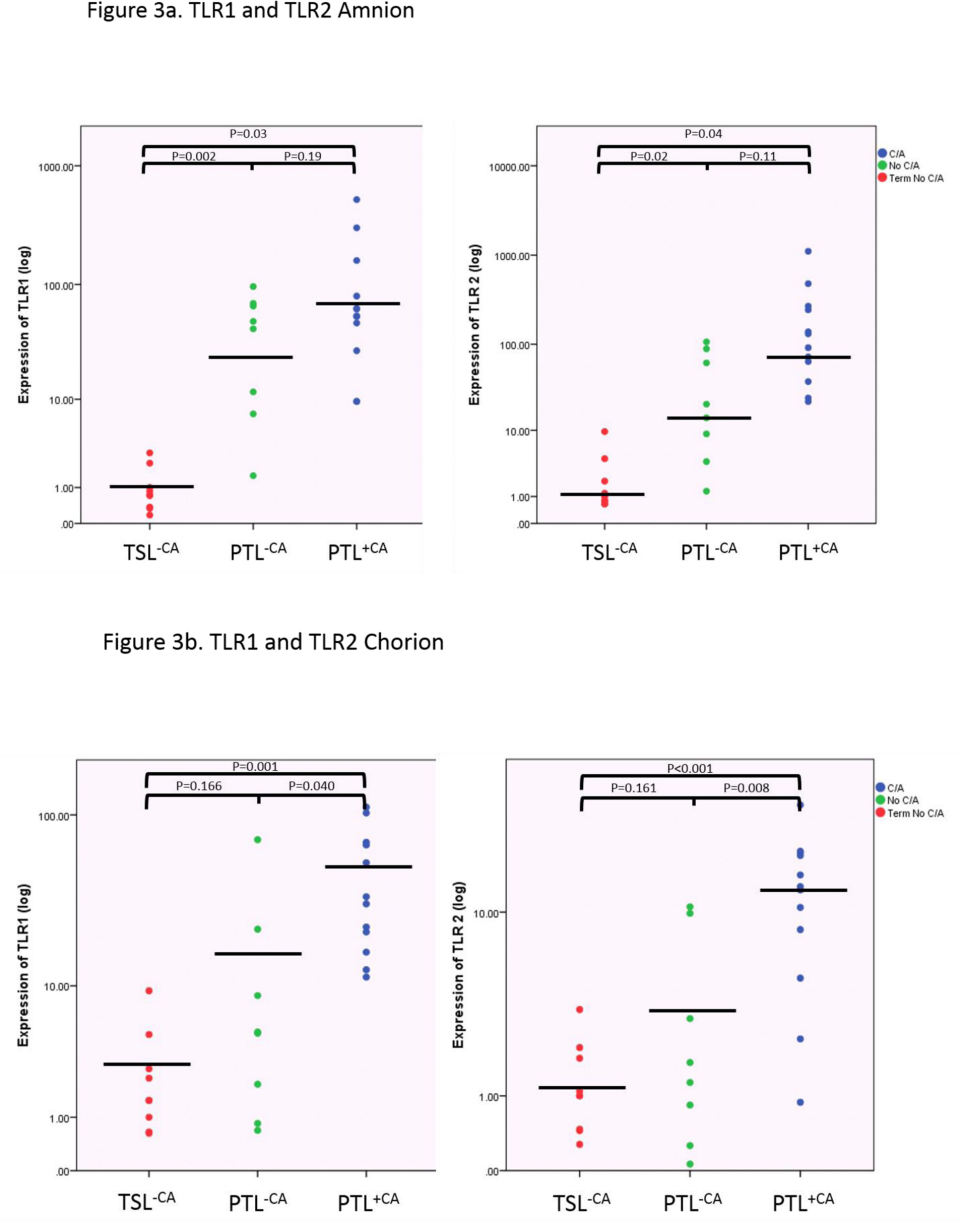
Fig 3a TLR expression in the amnion: Preterm labour with and without chorioamnionitis is associated with increased expression of TLR 1 and TLR 2 compared to term labour. All individual samples represented on plot, black line represents mean of samples. A significantly increased mean expression of TLR1and TLR 2 was noted in both the preterm birth groups when comparing to TSL^-CA^. (TLR1; PTL^+CA^ mean expression 82.58 vs TSL^-CA^; p = 0.035, PTL^-CA^ mean expression 32.73 vs TSL^-CA^; p = 0.002) (TLR2; PTL^+CA^ mean expression 80.33 vs TSL^-CA^; p = 0.046, PTL^-CA^ mean expression 12.04 vs TSL^-CA^; p = 0.020). Fig 3b TLR expression in the chorion: Histological chorioamnionitis is associated with increased expression of TLR 1 and TLR 2. All individual samples represented on plot, black line represents mean of samples. In the chorion the PTL^+CA^ group showed a significantly increased expression of TLR1 and TLR2. (TLR1; PTL^+CA^ mean expression 18.81 vs TSL^-CA^; p = 0.001, PTL^+CA^ mean expression 6.93 vs PTL^-CA^; p = 0.040) (TLR2; PTL^+CA^ mean expression 9.35 vs TSL^-CA^; p<0.001, PTL^+CA^ mean expression 6.93 vs PTL^-CA^; p = 0.008). Expression normalised to GapDH. Gene expression assessed by fold change (2^ΔΔCT^).

When comparing the preterm labour groups (PTL^+CA^ and PTL^-CA^), TLR 1, TLR 2 and LY96 showed increased expression with HCA (Fig 3) in the chorion. Both IL8 and IRAK2 showed increased expression in the amnion. The majority of the inflammatory genes showed a trend towards increased expression with HCA (Table 4).

**Table 4:** Gene expression: PTL^+CA^ vs PTL^-CA^.

Gene	Amnion	p	Chorion	p
**HMGB1**	-1.4171	0.351268	1.195	0.928029
**IL8**	35.2146	***0*.*04856***	29.7566	0.051431
**IRAK2**	8.0085	***0*.*026099***	11.0543	0.050549
**LY96**	2.5618	0.10761	10.2697	***0*.*002528***
**MyD88**	1.1651	0.282318	1.1852	0.691993
**SARM1**	-2.1468	0.164682	-1.7167	0.663633
**SIGIRR**	1.7599	0.686741	-1.2182	0.3628
**TIRAP**	-1.1721	0.846974	1.0561	0.603179
**TLR1**	2.5228	0.199452	6.9289	***0*.*0395***
**TLR2**	6.6696	0.111477	6.9289	***0*.*008355***
**TLR4**	2.0698	0.189518	1.1847	0.44966
**TLR6**	-2.5174	0.198552	1.3163	0.771412

Mean expression values shown. Students t-test used to test for significance (p<0.05). Expression normalised to GapDH. Gene expression assessed by fold change (2^ΔΔCT^).

**Fig 4:**
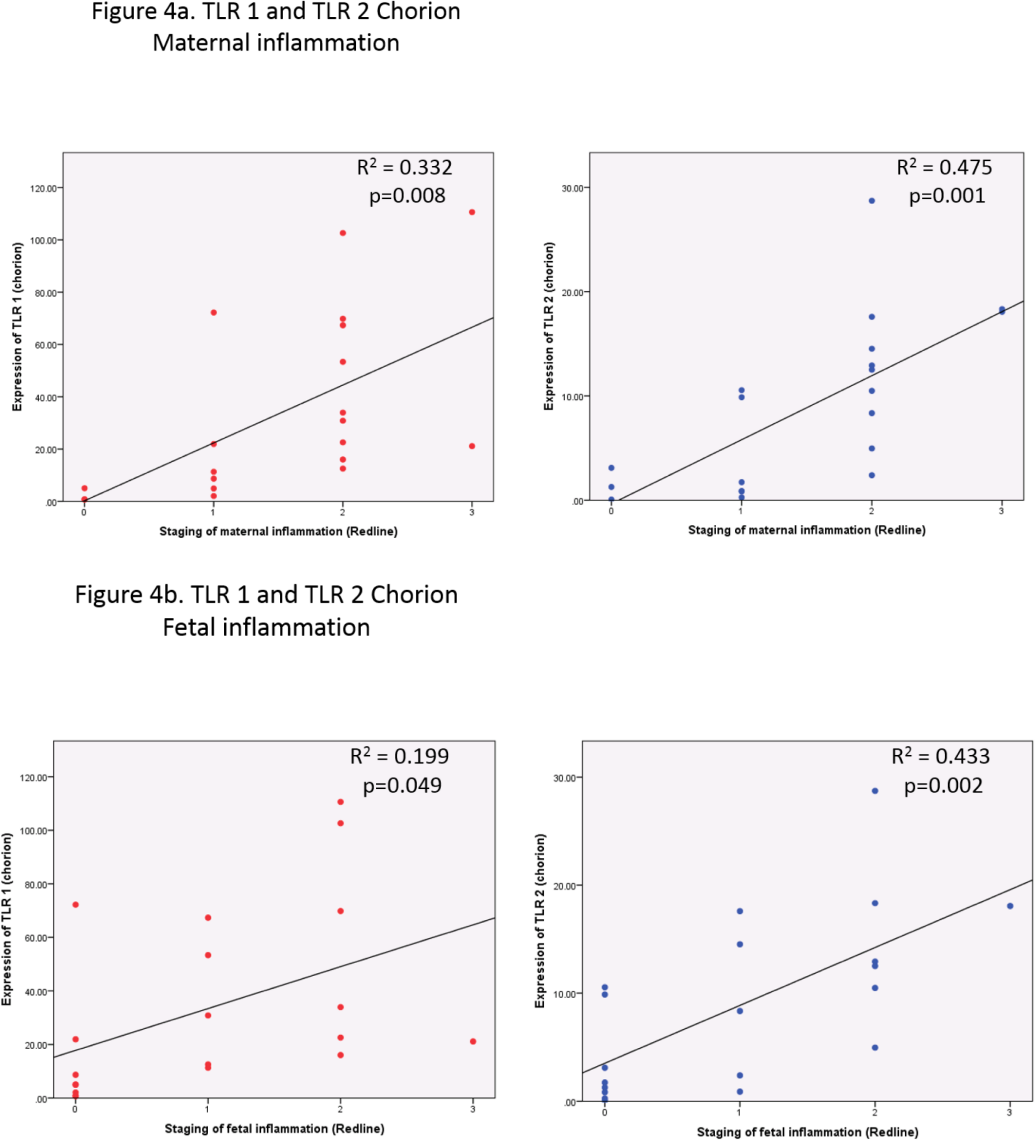
Severity of histological staging of chorioamnionitis correlates with expression of TLR 1 and TLR 2. This figure shows the relationship between expression of TLR 1 and TLR 2 in the chorion and staging of maternal (4a) and fetal (4b) inflammation. Least squares linear regression was used. All samples staged via Redline criteria included. Higher staging of maternal inflammation correlates with higher expression of TLR1 (R^2^ = 0.332; p = 0.008) and TLR 2 (R^2^ = 0.475; p = 0.001). Higher staging of fetal inflammation shows a similar pattern for TLR 1 (R^2^ = 0.199; p = 0.049) and TLR 2 (R^2^ = 0.433; p = 0.002). Expression normalised to GapDH. Gene expression assessed by fold change (2^ΔΔCT^).

We used linear regression to assess the relationship between gene expression and histological staging[22] in all preterm samples (S6 and S7 Tables). An increased maternal response and fetal response correlated with increased expression of TLR 1, TLR 2, LY96, IL8 and IRAK2 (p<0.05) in both amnion and chorion and of TLR 4 in the amnion. The data for TLR 1 and TLR 2 in the chorion is shown in Fig 4.

## Discussion

This is the first study to describe the involvement of TLR 1 in preterm histological chorioamnionitis (pHCA) separately in both amnion and chorion. Alongside TLR 1; TLR 2, LY96, IL8 and IRAK2 expression increased in pHCA when controlling for inflammation and gestational age. Increased expression of these genes, together with TLR 4, was found with higher degree of inflammation. Overall this increase in expression of multiple genes shows a heterogeneous inflammatory picture. It is important to put this picture into perspective clinically as the host response to pathogens is recognised as the primary event in the development of clinically significant chorioamnionitis[25]. The fetal inflammatory response (FIRS) is the pathway by which chorioamnionitis is associated with clinically significant sequelae[26]. Histologically, FIRS can be seen in the fetal inflammatory response. Our study shows this increased fetal response, via staging, correlates with increased expression of the above mentioned genes and therefore we infer their involvement in clinically significant pHCA.

TLR 1 is known to form heterodimers with TLR 2 on the surface of cell membranes allowing a far greater recognition of bacterial diversity. These heterodimers pre-exist and are not induced by the ligand. With relevance to this study TLR 1/TLR 2 heterodimers can be activated by bacterial triacylated lipoproteins [27] found in gram positive bacteria and genital mycoplasms. Expression of TLR 1 in pHCA has been examined in the membranes [28]. Reassuringly the authors also found pHCA was associated with increased expression of TLR 1 and TLR 2 when compared to preterm without HCA. As the authors did not separate amnion from chorion the results could not comment on the difference between each tissue. Our work has established this is present in the chorion in this setting with only a trend towards it in the amnion. In keeping with this, *Gillaux et al.[29]* attempted to model the effect of stimulation of TLR 1 and TLR 2 in amniotic epithelial cells but did not demonstrate an increase in inflammatory cytokines. TLR 1 has also been studied in other conditions; genetic variants of TLR 1 have been shown to increase susceptibility to complications of sepsis, leprosy, pelvic inflammatory disease and placental malaria [30–33]. In support of a role for TLR 1 in HCA, levels of the soluble form of TLR 1 (along with the soluble form of TLR 2 and 6) were found to be raised in amniotic fluid in cases of PTB with microbial invasion of the amniotic cavity[34]. Moreover, in gastrointestinal and intratracheal lipopolysacaride exposure in fetal sheep, designed to investigate chorioamnionitis-induced fetal gut injury, Kacerovsky et al. [31] reported mRNA upregulation of TLR 1 as well as TLR 2, 4 and 6[35].

We found pHCA was associated with increased expression of TLR 2 but not TLR 4 mRNA consistent with the findings of *Kim et al* [18]. However our study provides other evidence of TLR 4 involvement in pHCA. Although we found a trend for increased TLR 4 expression with pHCA we did find increased expression of the key accessory protein for LPS-induced TLR 4 signalling, LY96. LY96 cooperates with both TLR2 and TLR4 in the innate immune response. TLR interacting protein MyD88 acts via IRAK2 to activate NFкB. SARM1 is a negative regulator of MyD88 dependant TLR signalling Further the correlation between the degree of histological inflammation and increased expression of TLR 4, whilst not as strong as TLR 1, 2 and LY96, would also point towards association. Therefore it would appear that TLR 1, TLR 2 and TLR 4 may all appear to play a role in signalling in pHCA.

The explanation for the heterogeneous inflammatory profile generated by pHCA is likely due to the activation of specific TLRs by their ligands (both exogenous and endogenous). The exogenous ligands from a number of gram positive and negative bacteria from the lower genital tract, pathogenic mycoplasmas and microorganisms from the oral tract have all been associated with sPTB. Microbiological studies in sPTB have found problems determining the relevance of culture-proven infection, given the polymicrobial nature of sPTB. Jones et al. reported 2 or more bacterial species in over 60% of placentas and membranes collected following preterm birth [36]. Different types of microorganisms have been shown to have markedly different and distinct effects upon fetal membrane TLR expression patterns; *mycoplasma hominis* (but not *ureaplasma urealyticum*, *gardnerella vaginalis* or Group B Streptococcus) *has been associated with* increased expression of TLR 4, TLR 6 and TLR 8 mRNA in term membranes [37]. *Ureaplasma parvum* has been reported to upregulate TLR 1/ TLR 2 and TLR 6/ TLR 2 heterodimers [38] although Triantafilou et al. demonstrated involvement of TLR 6/ TLR 2 and TLR 9[39]. The heterogeneity of identified organisms and the inflammatory pattern generated makes tailoring potential interventions challenging. Variation may also be explained by TLRs reacting to endogenous ‘danger signals’ such as damage associated molecular patterns (DAMPs), released when cells are damaged, rather than simply by the presence of microorganisms, and both TLR 2 and TLR 4 have multiple known endogenous ligands [40].

In the setting of sPTB, it is the association with inflammation rather than culture-proven infection that correlates with both adverse outcomes and pathology[41]. Our study clearly shows that increased level of expression of TLR 1 and TLR 2 correlate with increased histological inflammation. Though the study numbers are too small to make any meaningful comment on the neonatal outcome data, the literature shows pHCA is clearly linked to the development of cerebral palsy and increased concentrations of proinflammatory cytokines in amniotic fluid and cord blood [42]. It stands to reason the prevention or amelioration of inflammation via TLR signalling could prove effective in both reduction in sPTB and poor neonatal outcomes. Indeed magnesium sulphate, a compound shown to be neuroprotective in the setting of preterm birth, has been shown to decrease TLR stimulated production of proinflammtory cytokines from neonatal monocytes [43].

Though TLR 1, TLR 2 and TLR 4 have distinct ligands, they activate a common pathway via the IRAK family and nuclear activation of transcription factor NFкB. Targeting downstream events in this pathway with immunomodulators has been shown to inhibit the proinflammatory cascade and resultant uterine activity in the non human primate model of sPTB [44–46]. This however may not protect the fetus from adverse sequelae associated with ascending infection. TLRs fulfil many of criteria regarded as essential to consider them therapeutic targets; they are over expressed in disease, knockout mice are resistant to disease, ligands exacerbate inflammation and genetic difference in TLRs correlate with risk of disease. Indeed outside of reproductive science, there are clinical phase trials using agonism/antagonism of TLR signalling in viral and bacterial infections, cancer and autoimmune disorders[13]. Further, in the setting of preterm birth TLR 4 antagonism inhibits LPS-induced uterine contractility and proinflammatory cascade in pregnant rhesus monkeys [47]. Antagonism of TLR 1 and TLR 2 in the setting of ascending infection in the fetal membranes has not yet been assessed. Our findings provide the first evidence that signalling events via these receptors are likely to be required for the resulting inflammation seen in pHCA. While the results need confirmation, our findings suggest an intervention focusing on a single TLR is unlikely to be successful in this setting.

Though we are confident that our results are robust there are some potential limitations to our study. The limitations relate to the difficulty of defining a preterm control group. The phenotyping of the preterm groups was based on the presence or absence of histological inflammation. Whilst samples from PTL^-CA^ did not display HCA there were other placental findings such as features of uteroplacental insufficiency, ischaemia and villitus. Given that sPTB is not a normal process we did anticipate that though PTL^-CA^ would not have HCA they also would not be ‘normal’ and we accepted this would be a limitation. We also considered lower stages of histological inflammation The stage of maternal inflammation at which point HCA is stated to present is stage 2[22]. As you can see from Fig 4, study samples showing stage 1 inflammation (subchorionitis) and included in PTL^-CA^, displayed higher levels of TLR 1 expression than those with a complete absence of histological inflammation. The inclusion of these samples in PTL^-CA^ can make conclusions based on comparisons of this group with TSL^-CA^ less reliable.Our study was the first to consider the expression of TLR signalling in both the amnion and chorion separately in pHCA. The fetal membranes are composed of these distinct layers. The amnion comprises a single layer of cuboidal epithelial cells and a thin layer of connective tissue. The chorion is comprised of somatic mesoderm (in contact with the amnion) and extra villous trophoblast. In our study the pattern of expression was similar in both tissues regardless of the group. However, when comparing the 2 preterm groups it was in the chorion that expression of TLR 1, TLR 2 and LY96 was significantly increased with pHCA. There is debate in the literature about the precise order in which the series of events transpire in ascending infection. This relates the initial focus of infection. It has been argued that process of chorioamnionitis, as seen via the histological staging, is a response to infection in the amnionitic fluid with associated amniotrophism (diffuse infiltration of maternal neutrophils from decidua towards the amniotic cavity). This view has been supported by work showing a higher 16srRNA gene copy number in the amnion when compared to the chorion for each stage of chorioamnionitis [48]. This would suggest that the organisms ascend, cross the membranes without causing an inflammatory response until the amniotic fluid is involved. Alternatively, it has also been argued that the inflammatory response is generated in response to bacterial invasion of the choriodecidual space secondary to colonisation of the uterus[49]. Colonisation of the uterus may precede pregnancy and only when the endometrial cavity is sealed by the expanding membranes in mid pregnancy does this infection become symptomatic. This raises the question as which of the fetal membranes is initially involved in ascending infection and highlights the importance of considering both.

## Supporting Information

S1 Table Signalling array analysis: PTL^+CA^ vs TSL^-CA^. Mean expression values shown. Students t-test used to test for significance (p<0.05). Expression normalised to GapDH. Gene expression assessed by fold change (2^ΔΔCT^).
(DOCX)

S2 Table Signalling array analysis: PTL^-CA^ vs TSL^-CA^. Mean expression values shown. Students t-test used to test for significance (p<0.05). Expression normalised to GapDH. Gene expression assessed by fold change (2^ΔΔCT^).
(DOCX)

S3 Table Signalling array analysis: PTL^+CA^ vs PTL^-CA^. Mean expression values shown. Students t-test used to test for significance (p<0.05). Expression normalised to GapDH. Gene expression assessed by fold change (2^ΔΔCT^).
(DOCX)

S4 Table Gene expression: PTL^-CA^ vs TSL^-CA^. Mean expression values shown. Students t-test used to test for significance (p<0.05). Expression normalised to GapDH. Gene expression assessed by fold change (2^ΔΔCT^).
(DOCX)

S5 Table The relationship between gene expression and gestational age without inflammation (PTL^-CA^ and TSL^-CA^). Least squares linear regression (p<0.05) was used. Expression normalised to GapDH. Gene expression assessed by fold change (2^ΔΔCT^).
(DOCX)

S6 Table The relationship between gene expression and histological staging (maternal). All samples from PTL^+CA^ and PTL^-CA^ were examined. Least squares linear regression (p<0.05) was used. Expression normalised to GapDH. Gene expression assessed by fold change (2^ΔΔCT^).
(DOCX)

S7 Table The relationship between gene expression and histological staging (fetal). All samples from PTL^+CA^ and PTL^-CA^ were examined. Least squares linear regression (p<0.05) was used. Expression normalised to GapDH. Gene expression assessed by fold change (2^ΔΔCT^).
(DOCX)
