# Supplementary material for: Inflammatory Signalling in Fetal Membranes: Increased Expression Levels of TLR 1 in the Presence of Preterm Histological Chorioamnionitis
Source: PLoS One. 2015 May 12;10(5):e0124298. doi: 10.1371/journal.pone.0124298 (PMC4429010; doi:10.1371/journal.pone.0124298)
Supplement: S1 Table — Mean expression values shown. Students t-test used to test for significance (p<0.05). Expression normalised to GapDH. Gene expression assessed by fold change (2ΔΔCT). (DOCX) [file pone.0124298.s001.docx]

S1 Table . Signalling array analysis: PTL^+CA^ vs TSL^-CA^.

| **Gene** | **Amnion** | **p** | **Chorion** | **p** |
| --- | --- | --- | --- | --- |
| BTK | 2.8846 | 0.381417 | 2.7735 | 0.318035 |
| CASP8 | -2.3128 | 0.776027 | 1.0698 | 0.523766 |
| CCL2 | 3.9362 | 0.346417 | 13.1525 | 0.169567 |
| CD14 | 5.9645 | 0.403948 | 4.8636 | 0.359416 |
| CD80 | 2.3714 | 0.386298 | -1.1826 | 0.64991 |
| CD86 | 1.396 | 0.517161 | 5.3685 | 0.399099 |
| CHUK | -17.6838 | 0.231625 | -3.378 | 0.400356 |
| CLEC4E | 137.4722 | 0.186657 | 454.4133 | 0.330487 |
| CSF2 | 1.4165 | 0.932752 | 44.7044 | 0.369846 |
| CSF3 | 1.1564 | 0.914047 | 7.5566 | 0.154517 |
| CXCL10 | 4.9936 | 0.37453 | 11.4312 | 0.19451 |
| EIF2AK2 | -1.4569 | 0.833299 | 1.1464 | 0.830694 |
| ELK1 | 2.7226 | 0.37767 | 1.0832 | 0.790153 |
| FADD | -18.1135 | 0.304412 | 2.0181 | 0.585492 |
| FOS | -6.8664 | 0.320054 | -10.4195 | 0.268252 |
| HMGB1 | -24.4697 | 0.108559 | -5.0843 | 0.068755 |
| HRAS | -13.475 | 0.291982 | -3.4246 | 0.175881 |
| HSPA1A | -4.3855 | 0.171771 | -1.0623 | 0.777105 |
| HSPD1 | -11.99 | 0.134875 | -2.2879 | 0.365724 |
| IFNA1 | -4.9139 | 0.324121 | 1.2249 | 0.844762 |
| IFNB1 | -6.1044 | 0.18404 | 2.3174 | 0.501216 |
| IFNG | -1.5764 | 0.615166 | -1.717 | 0.641147 |
| IKBKB | -5.4315 | 0.148226 | 2.185 | 0.336107 |
| IL10 | 4.3217 | 0.123282 | 10.6231 | 0.228023 |
| IL12A | -1.5349 | 0.520271 | 1.4555 | 0.862829 |
| IL1A | 6.0368 | 0.138708 | 293.0345 | 0.297429 |
| IL1B | 124.7991 | 0.103763 | 293.4973 | 0.185163 |
| IL2 | -3.6463 | 0.370269 | 3.0553 | 0.42987 |
| IL6 | 9.3675 | 0.151294 | 19.4555 | 0.254349 |
| IL8 | 34.2008 | 0.001272 | 258.4951 | 0.329207 |
| IRAK1 | -2.2779 | 0.308419 | 1.552 | 0.80249 |
| IRAK2 | 3.9632 | 0.269285 | 45.768 | 0.294201 |
| IRF1 | 1.2008 | 0.574434 | 7.4394 | 0.290522 |
| IRF3 | -10.8826 | 0.315915 | -4.1595 | 0.265962 |
| JUN | 8.9841 | 0.272158 | 27.0164 | 0.128299 |
| LTA | -1.2818 | 0.455033 | 2.7675 | 0.285754 |
| CD180 | 2.7456 | 0.380877 | 1.4238 | 0.821491 |
| LY86 | 2.358 | 0.321727 | -1.9024 | 0.379715 |
| LY96 | 61.1382 | 0.035744 | 29.1309 | 0.08731 |
| MAP2K3 | -2.9702 | 0.207388 | 1.3993 | 0.618419 |
| MAP2K4 | -34.4867 | 0.225092 | -11.6212 | 0.221476 |
| MAP3K1 | -38.2687 | 0.218302 | -7.7758 | 0.348355 |
| MAP3K7 | -13.7728 | 0.246783 | -8.2041 | 0.286422 |
| TAB1 | -3.5459 | 0.325594 | -4.3767 | 0.30865 |
| MAP4K4 | -2.5071 | 0.310783 | 5.0806 | 0.441168 |
| MAPK8 | 3.5445 | 0.520443 | 2.7158 | 0.688002 |
| MAPK8IP3 | -1.3526 | 0.366604 | 2.3714 | 0.953366 |
| MYD88 | 2.2285 | 0.083901 | -1.9513 | 0.717565 |
| NFKB1 | 3.6363 | 0.328302 | 8.533 | 0.936573 |
| NFKB2 | -3.1476 | 0.892567 | 1.2986 | 0.497258 |
| NFKBIA | 1.2224 | 0.633729 | 4.4423 | 0.148447 |
| NFKBIL1 | -2.1732 | 0.551351 | 1.7459 | 0.453888 |
| NFRKB | -11.4096 | 0.235172 | -4.3474 | 0.278234 |
| NR2C2 | -18.6146 | 0.20866 | -16.9335 | 0.178473 |
| PELI1 | -3.0053 | 0.424229 | 2.6964 | 0.617394 |
| PPARA | -16.7191 | 0.30599 | -1.0501 | 0.783719 |
| PRKRA | -10.5616 | 0.293032 | 2.0067 | 0.893011 |
| PTGS2 | -3.1581 | 0.379201 | 3.7709 | 0.414152 |
| REL | 66.8262 | 0.264958 | 166.3202 | 0.11351 |
| RELA | 1.7655 | 0.58403 | 4.2836 | 0.343289 |
| RIPK2 | -1.3088 | 0.587976 | 5.7368 | 0.323227 |
| SARM1 | -2.6417 | 0.061882 | 1.5696 | 0.705818 |
| SIGIRR | -1.1029 | 0.99483 | 1.0686 | 0.628477 |
| ECSIT | -2.8361 | 0.378588 | 2.4805 | 0.197093 |
| TBK1 | -3.2516 | 0.290305 | -4.0932 | 0.352097 |
| TICAM2 | -3.4194 | 0.317384 | -3.9026 | 0.449109 |
| TIRAP | -15.5079 | 0.296238 | -2.9485 | 0.41718 |
| TLR1 | 5.1494 | 0.437923 | 5.105 | 0.090694 |
| TLR10 | -1.1545 | 0.470435 | 2.2244 | 0.387953 |
| TLR2 | 39.2705 | 0.091239 | 150.9267 | 0.078729 |
| TLR3 | 5.3365 | 0.319515 | 4.5909 | 0.460011 |
| TLR4 | 22.6248 | 0.090627 | 4.9486 | 0.277015 |
| TLR5 | -2.7063 | 0.539192 | 1.4313 | 0.831061 |
| TLR6 | 2.5175 | 0.078611 | 1.5344 | 0.766696 |
| TLR7 | 2.0945 | 0.222858 | 1.4328 | 0.674954 |
| TLR8 | 7.0539 | 0.14049 | -1.089 | 0.717386 |
| TLR9 | -1.0127 | 0.775563 | 3.0695 | 0.1019 |
| TNF | 4.6607 | 0.425639 | 10.9495 | 0.178823 |
| TNFRSF1A | -6.4219 | 0.290985 | -2.8839 | 0.36836 |
| TOLLIP | -9.2127 | 0.330215 | -4.0753 | 0.258972 |
| TRAF6 | -5.6995 | 0.366534 | -2.1513 | 0.337062 |
| TICAM1 | -3.8202 | 0.37113 | 1.94 | 0.432135 |
| UBE2N | 5.6164 | 0.471993 | 3.0799 | 0.934726 |
| UBE2V1 | -1.3052 | 0.847356 | 1.9921 | 0.314492 |

Mean expression values shown. Students t-test used to test for significance (p<0.05). Expression normalised to GapDH. Gene expression assessed by fold change (2^ΔΔCT^).
