# Supplementary material for: Inflammatory Signalling in Fetal Membranes: Increased Expression Levels of TLR 1 in the Presence of Preterm Histological Chorioamnionitis
Source: PLoS One. 2015 May 12;10(5):e0124298. doi: 10.1371/journal.pone.0124298 (PMC4429010; doi:10.1371/journal.pone.0124298)
Supplement: S2 Table — Mean expression values shown. Students t-test used to test for significance (p<0.05). Expression normalised to GapDH. Gene expression assessed by fold change (2ΔΔCT). (DOCX) [file pone.0124298.s002.docx]

S2 Table. Signalling array analysis: PTL^-CA^ vs TSL^-CA^.

| **Gene** | **Amnion** | **p** | **Chorion** | **p** |
| --- | --- | --- | --- | --- |
| ELK1 | 1.1996 | 0.946022 | 2.2805 | 0.360873 |
| FADD | -22.8659 | 0.28305 | 1.5029 | 0.969454 |
| FOS | -2.0749 | 0.400226 | -8.0092 | 0.11827 |
| HMGB1 | -5.6161 | 0.134719 | -5.5015 | 0.029125 |
| HRAS | -8.9657 | 0.292533 | -5.8463 | 0.128694 |
| HSPA1A | -6.1312 | 0.225441 | -11.5908 | 0.169349 |
| HSPD1 | -14.7662 | 0.129697 | -11.5058 | 0.238009 |
| IFNA1 | -11.1353 | 0.332623 | -13.0385 | 0.235838 |
| IFNB1 | -3.0127 | 0.434734 | -11.5407 | 0.239308 |
| IFNG | -1.7075 | 0.468997 | -29.5135 | 0.172968 |
| IKBKB | -6.34 | 0.204733 | -3.1652 | 0.209951 |
| IL10 | 1.1184 | 0.821811 | -5.1484 | 0.185619 |
| IL12A | -6.4232 | 0.570273 | -10.9039 | 0.265292 |
| IL1A | -1.6292 | 0.457093 | 4.7386 | 0.840675 |
| IL1B | -1.0558 | 0.375824 | 1.177 | 0.912079 |
| IL2 | -14.2719 | 0.36636 | -16.6629 | 0.2656 |
| IL6 | -1.6979 | 0.585695 | -11.5531 | 0.307434 |
| IL8 | -4.7536 | 0.630464 | -5.2682 | 0.30672 |
| IRAK1 | -9.1224 | 0.22833 | -14.7857 | 0.320716 |
| IRAK2 | -7.4744 | 0.237605 | -8.2775 | 0.006821 |
| IRF1 | -2.1154 | 0.655297 | -4.7044 | 0.357103 |
| IRF3 | -4.1305 | 0.34602 | -12.0339 | 0.210718 |
| JUN | 6.8981 | 0.451689 | 2.3095 | 0.663431 |
| LTA | 1.2462 | 0.813741 | -16.2598 | 0.132395 |
| CD180 | 2.6787 | 0.31557 | -3.3151 | 0.50924 |
| LY86 | 2.3149 | 0.44811 | -2.6634 | 0.823636 |
| LY96 | 6.2361 | 0.244593 | -2.4446 | 0.441812 |
| MAP2K3 | -3.501 | 0.199971 | -7.578 | 0.138477 |
| MAP2K4 | -16.2775 | 0.230232 | -10.7717 | 0.214299 |
| MAP3K1 | -10.8573 | 0.221947 | -18.2179 | 0.338653 |
| MAP3K7 | -13.7526 | 0.240932 | -15.2134 | 0.270486 |
| TAB1 | -2.3143 | 0.499334 | -5.3589 | 0.310209 |
| MAP4K4 | -9.0134 | 0.215512 | -4.2894 | 0.364293 |
| MAPK8 | -1.2229 | 0.803666 | -1.4474 | 0.447293 |
| MAPK8IP3 | -1.1394 | 0.327768 | -4.4526 | 0.305183 |
| MYD88 | -3.3326 | 0.269349 | -10.571 | 0.273743 |
| NFKB1 | 1.7468 | 0.649381 | 5.7434 | 0.698281 |
| NFKB2 | -1.2999 | 0.673801 | -1.0715 | 0.579615 |
| NFKBIA | -2.8278 | 0.236237 | -1.765 | 0.363059 |
| NFKBIL1 | -1.19 | 0.955325 | 4.4841 | 0.110134 |
| NFRKB | -1.9063 | 0.403323 | -3.4369 | 0.279063 |
| NR2C2 | -7.5251 | 0.232742 | -9.2637 | 0.23372 |
| PELI1 | -5.3708 | 0.287625 | -10.6242 | 0.300523 |
| PPARA | -5.5065 | 0.32281 | -4.9286 | 0.1654 |
| PRKRA | -7.5451 | 0.2794 | -1.0138 | 0.347213 |
| PTGS2 | -12.0653 | 0.360174 | -22.9216 | 0.245953 |
| REL | 14.3561 | 0.915126 | 2.0452 | 0.612205 |
| RELA | -3.1532 | 0.282842 | -8.3244 | 0.268275 |
| RIPK2 | -1.2092 | 0.813244 | 4.3581 | 0.194854 |
| SARM1 | -1.4683 | 0.386097 | -1.1746 | 0.626966 |
| SIGIRR | -1.2351 | 0.548926 | -5.266 | 0.339932 |
| ECSIT | -2.3382 | 0.410434 | -2.2223 | 0.282287 |
| TBK1 | -2.2698 | 0.385306 | -8.1441 | 0.260566 |
| TICAM2 | -4.9685 | 0.320886 | -12.9803 | 0.170193 |
| TIRAP | -7.4529 | 0.075979 | -14.0416 | 0.277494 |
| TLR1 | -2.5403 | 0.479858 | -10.9104 | 0.282819 |
| TLR10 | -10.1744 | 0.390858 | -26.6171 | 0.526482 |
| TLR2 | 1.8602 | 0.380569 | -3.1981 | 0.336666 |
| TLR3 | 2.3105 | 0.851291 | -1.0938 | 0.738287 |
| TLR4 | 1.1271 | 0.549153 | -7.4685 | 0.248578 |
| TLR5 | -1.6427 | 0.405253 | 2.1757 | 0.582885 |
| TLR6 | -1.2087 | 0.444168 | 2.0751 | 0.811998 |
| TLR7 | 1.1464 | 0.394687 | -2.371 | 0.042859 |
| TLR8 | 1.8712 | 0.244245 | -13.0713 | 0.265035 |
| TLR9 | -1.6015 | 0.565006 | -5.033 | 0.137434 |
| TNF | -1.5905 | 0.980311 | -4.7694 | 0.308756 |
| TNFRSF1A | -5.8867 | 0.290166 | -8.3695 | 0.239689 |
| TOLLIP | -2.3718 | 0.387974 | -4.0839 | 0.250433 |
| TRAF6 | -3.1675 | 0.387331 | -4.8964 | 0.226493 |
| TICAM1 | -2.6141 | 0.395355 | -4.6318 | 0.232507 |
| UBE2N | 4.5978 | 0.453135 | -2.8336 | 0.383735 |
| UBE2V1 | -1.4413 | 0.541442 | -7.0764 | 0.199328 |

Mean expression values shown. Students t-test used to test for significance (p<0.05). Expression normalised to GapDH. Gene expression assessed by fold change (2^ΔΔCT^).
