# Supplementary material for: Inflammatory Signalling in Fetal Membranes: Increased Expression Levels of TLR 1 in the Presence of Preterm Histological Chorioamnionitis
Source: PLoS One. 2015 May 12;10(5):e0124298. doi: 10.1371/journal.pone.0124298 (PMC4429010; doi:10.1371/journal.pone.0124298)
Supplement: S3 Table — Mean expression values shown. Students t-test used to test for significance (p<0.05). Expression normalised to GapDH. Gene expression assessed by fold change (2ΔΔCT). (DOCX) [file pone.0124298.s003.docx]

S3 Table. Signalling array analysis: PTL^+CA^ vs PTL^-CA^.

| **Gene** | **Amnion** | **p** | **Chorion** | **p** |
| --- | --- | --- | --- | --- |
| BTK | 6.4759 | 0.386235 | 14.5519 | 0.27965 |
| CASP8 | -1.8195 | 0.984189 | -1.1382 | 0.602789 |
| CCL2 | 10.8152 | 0.247675 | 25.4663 | 0.162165 |
| CD14 | 8.7364 | 0.227631 | 71.1167 | 0.336287 |
| CD80 | 30.9848 | 0.369348 | 3.5807 | 0.358558 |
| CD86 | 6.5015 | 0.158005 | 31.1659 | 0.243577 |
| CHUK | 1.2485 | 0.704329 | 5.2926 | 0.254635 |
| CLEC4E | 260.5019 | 0.16057 | 806.0506 | 0.330378 |
| CSF2 | 4.8372 | 0.360437 | 158.3519 | 0.128593 |
| CSF3 | 24.4817 | 0.370443 | 26.7538 | 0.127255 |
| CXCL10 | 34.3445 | 0.37389 | 73.9024 | 0.136687 |
| EIF2AK2 | 2.3302 | 0.391442 | 10.3146 | 0.111928 |
| ELK1 | 2.2696 | 0.376374 | -2.1054 | 0.154121 |
| FADD | 1.2624 | 0.552347 | 1.3428 | 0.583125 |
| FOS | -3.3092 | 0.323418 | -1.3009 | 0.709205 |
| HMGB1 | -4.3571 | 0.331829 | 1.0821 | 0.611574 |
| HRAS | -1.503 | 0.959323 | 1.7071 | 0.269815 |
| HSPA1A | 1.398 | 0.876289 | 10.9107 | 0.174923 |
| HSPD1 | 1.2315 | 0.867415 | 5.0289 | 0.215893 |
| IFNA1 | 2.2661 | 0.92942 | 15.9703 | 0.124248 |
| IFNB1 | -2.0263 | 0.378767 | 26.7449 | 0.187953 |
| IFNG | 1.0832 | 0.650311 | 17.189 | 0.106424 |
| IKBKB | 1.1673 | 0.655878 | 6.916 | 0.243046 |
| IL10 | 3.864 | 0.258796 | 54.6917 | 0.209555 |
| IL12A | 4.1849 | 0.923943 | 15.8705 | 0.119113 |
| IL1A | 9.8354 | 0.117895 | 61.8399 | 0.297786 |
| IL1B | 131.7577 | 0.106605 | 249.3663 | 0.185192 |
| IL2 | 3.9141 | 0.912128 | 50.9107 | 0.252964 |
| IL6 | 15.9048 | 0.197445 | 224.7712 | 0.232865 |
| IL8 | 162.5768 | 0.003822 | 1361.7975 | 0.328346 |
| IRAK1 | 4.0048 | 0.145354 | 22.9479 | 0.204554 |
| IRAK2 | 29.6227 | 0.039828 | 378.8429 | 0.290543 |
| IRF1 | 2.5401 | 0.469527 | 34.9978 | 0.130543 |
| IRF3 | -2.6347 | 0.268571 | 2.8931 | 0.296885 |
| JUN | 1.3024 | 0.462145 | 11.698 | 0.210904 |
| LTA | -1.5973 | 0.44349 | 44.9984 | 0.185799 |
| CD180 | 1.025 | 0.437745 | 4.7199 | 0.364996 |
| LY86 | 1.0186 | 0.935271 | 1.4 | 0.490423 |
| LY96 | 9.8039 | 0.052066 | 71.2145 | 0.085068 |
| MAP2K3 | 1.1787 | 0.889086 | 10.6042 | 0.218227 |
| MAP2K4 | -2.1187 | 0.487211 | -1.0789 | 0.729333 |
| MAP3K1 | -3.5247 | 0.676799 | 2.3429 | 0.402856 |
| MAP3K7 | -1.0015 | 0.667287 | 1.8544 | 0.318301 |
| TAB1 | -1.5322 | 0.508654 | 1.2244 | 0.966988 |
| MAP4K4 | 3.5951 | 0.165542 | 21.7927 | 0.290801 |
| MAPK8 | 4.3346 | 0.381713 | 3.9307 | 0.43189 |
| MAPK8IP3 | -1.1871 | 0.883845 | 10.5589 | 0.161641 |
| MYD88 | 1.7079 | 0.659479 | 23.5581 | 0.21255 |
| NFKB1 | 2.0817 | 0.396051 | 1.4857 | 0.45659 |
| NFKB2 | -2.4214 | 0.906867 | 1.3915 | 0.426706 |
| NFKBIA | 3.4566 | 0.278954 | 7.8408 | 0.10775 |
| NFKBIL1 | -1.8262 | 0.659735 | -2.5683 | 0.296597 |
| NFRKB | -5.9854 | 0.396063 | -1.2649 | 0.9771 |
| NR2C2 | -2.4737 | 0.363406 | -1.8279 | 0.313193 |
| PELI1 | 1.7871 | 0.492082 | 28.6468 | 0.184441 |
| PPARA | -3.0363 | 0.247783 | 4.6933 | 0.105728 |
| PRKRA | -1.3998 | 0.736303 | 2.0344 | 0.406313 |
| PTGS2 | 3.8204 | 0.211435 | 86.4353 | 0.257406 |
| REL | 4.6549 | 0.265457 | 81.3235 | 0.101818 |
| RELA | 5.5669 | 0.3196 | 35.6586 | 0.125539 |
| RIPK2 | -1.0823 | 0.649664 | 1.3164 | 0.523209 |
| SARM1 | -1.7992 | 0.37643 | 1.8437 | 0.716796 |
| SIGIRR | 1.1198 | 0.563977 | 5.6271 | 0.06139 |
| ECSIT | -1.213 | 0.827067 | 5.5125 | 0.10514 |
| TBK1 | -1.4326 | 0.64243 | 1.9897 | 0.396311 |
| TICAM2 | 1.453 | 0.954317 | 3.3261 | 0.315238 |
| TIRAP | -2.0808 | 0.350976 | 4.7623 | 0.356954 |
| TLR1 | 13.0811 | 0.118918 | 55.6981 | 0.229421 |
| TLR10 | 8.813 | 0.387317 | 59.2082 | 0.262855 |
| TLR2 | 21.1114 | 0.146643 | 482.6858 | 0.077489 |
| TLR3 | 2.3097 | 0.29953 | 5.0216 | 0.297034 |
| TLR4 | 20.0732 | 0.062835 | 36.9586 | 0.227942 |
| TLR5 | -1.6475 | 0.58095 | -1.5201 | 0.382991 |
| TLR6 | 3.043 | 0.194548 | -1.3524 | 0.45624 |
| TLR7 | 1.8271 | 0.642604 | 3.3972 | 0.285651 |
| TLR8 | 3.7696 | 0.176842 | 12.0031 | 0.217109 |
| TLR9 | 1.5813 | 0.693697 | 15.4486 | 0.125603 |
| TNF | 7.4129 | 0.429443 | 52.2222 | 0.158519 |
| TNFRSF1A | -1.0909 | 0.952864 | 2.9022 | 0.161868 |
| TOLLIP | -3.8843 | 0.274781 | 1.0021 | 0.852356 |
| TRAF6 | -1.7994 | 0.881825 | 2.276 | 0.253098 |
| TICAM1 | -1.4614 | 0.896241 | 8.9859 | 0.194052 |
| UBE2N | 1.2215 | 0.864062 | 8.7271 | 0.202359 |
| UBE2V1 | 1.1043 | 0.527825 | 14.0969 | 0.131224 |

Mean expression values shown. Students t-test used to test for significance (p<0.05). Expression normalised to GapDH. Gene expression assessed by fold change (2^ΔΔCT^).
