# Supplementary material for: Inflammatory Signalling in Fetal Membranes: Increased Expression Levels of TLR 1 in the Presence of Preterm Histological Chorioamnionitis
Source: PLoS One. 2015 May 12;10(5):e0124298. doi: 10.1371/journal.pone.0124298 (PMC4429010; doi:10.1371/journal.pone.0124298)
Supplement: S4 Table — Mean expression values shown. Students t-test used to test for significance (p<0.05). Expression normalised to GapDH. Gene expression assessed by fold change (2ΔΔCT). (DOCX) [file pone.0124298.s004.docx]

S4 Table. Gene expression: PTL^-CA^ vs TSL^-CA^.

| **Gene** | **Amnion** | **p** | **Chorion** | **p** |
| --- | --- | --- | --- | --- |
| **HMGB1** | 1.1518 | 0.49201 | -1.0721 | 0.878665 |
| **IL8** | 9.0835 | 0.187685 | 1.8372 | 0.166163 |
| **IRAK2** | 13.7091 | ***0.006223*** | 2.5279 | 0.123489 |
| **LY96** | 41.1614 | ***0.002079*** | 2.531 | 0.108016 |
| **MyD88** | 2.5183 | ***0.008556*** | -1.1244 | 0.554399 |
| **SARM1** | 8.8398 | ***0.00336*** | 1.7597 | 0.282105 |
| **SIGIRR** | 1.8952 | 0.176056 | 2.6095 | 0.313799 |
| **TIRAP** | 2.2281 | 0.133797 | 1.0154 | 0.476911 |
| **TLR1** | 32.7338 | ***0.002103*** | 2.7149 | 0.165933 |
| **TLR2** | 12.0438 | ***0.020083*** | 1.3491 | 0.161438 |
| **TLR4** | 18.3764 | ***0.007841*** | -1.0121 | 0.802086 |
| **TLR6** | 5.9509 | 0.247784 | 1.2599 | 0.311314 |

Mean expression values shown. Students t-test used to test for significance (p<0.05). Expression normalised to GapDH. Gene expression assessed by fold change (2^ΔΔCT^).
