# Supplementary material for: Inflammatory Signalling in Fetal Membranes: Increased Expression Levels of TLR 1 in the Presence of Preterm Histological Chorioamnionitis
Source: PLoS One. 2015 May 12;10(5):e0124298. doi: 10.1371/journal.pone.0124298 (PMC4429010; doi:10.1371/journal.pone.0124298)
Supplement: S5 Table — Least squares linear regression (p<0.05) was used. Expression normalised to GapDH. Gene expression assessed by fold change (2ΔΔCT). (DOCX) [file pone.0124298.s005.docx]

S5 Table. The relationship between gene expression and gestational age without inflammation (PTL^-CA^ and TSL^-CA^).

| **Gene** | **R^2^ (amnion)** | **P value** | **R^2^ (chorion)** | **P value** |
| --- | --- | --- | --- | --- |
| TLR 1 | 0.671 | ***<0.001*** | 0.411 | ***0.006*** |
| TLR 2 | 0.163 | 0.108 | 0.318 | ***0.018*** |
| TLR 4 | 0.523 | ***0.001*** | 0.000 | 0.981 |
| TLR 6 | 0.019 | 0.600 | 0.313 | ***0.019*** |
| SARM1 | 0.376 | ***0.010*** | 0.015 | 0.634 |
| MyD88 | 0.456 | ***0.003*** | 0.003* | 0.831 |
| LY96 | 0.628 | ***<0.001*** | 0.473 | ***0.002*** |
| IL8 | 0.025 | 0.546 | 0.283 | ***0.028*** |
| IRAK2 | 0.240 | ***0.046*** | 0.415 | ***0.005*** |
| HMGB1 | 0.018 | 0.607 | 0.095 | 0.229 |
| SIGIRR | 0.369 | ***0.028*** | 0.309 | ***0.039*** |
| TIRAP | 0.458 | ***0.003*** | 0.126 | 0.163 |

Least squares linear regression (p<0.05) was used. Expression normalised to GapDH. Gene expression assessed by fold change (2^ΔΔCT^).
