# Supplementary material for: Inflammatory Signalling in Fetal Membranes: Increased Expression Levels of TLR 1 in the Presence of Preterm Histological Chorioamnionitis
Source: PLoS One. 2015 May 12;10(5):e0124298. doi: 10.1371/journal.pone.0124298 (PMC4429010; doi:10.1371/journal.pone.0124298)
Supplement: S6 Table — All samples from PTL+CA and PTL-CA were examined. Least squares linear regression (p<0.05) was used. Expression normalised to GapDH. Gene expression assessed by fold change (2ΔΔCT). (DOCX) [file pone.0124298.s006.docx]

S6 Table. The relationship between gene expression and histological staging (maternal).

| **Gene** | **R^2^ (amnion)** | **P value** | **R^2^ (chorion)** | **P value** |
| --- | --- | --- | --- | --- |
| TLR 1 | 0.371 | ***0.004*** | 0.332 | ***0.008*** |
| TLR 2 | 0.380 | ***0.004*** | 0.475 | ***0.001*** |
| TLR 4 | 0.368 | ***0.005*** | 0.030 | 0.467 |
| TLR 6 | 0.166* | 0.074 | 0.001* | 0.876 |
| SARM1 | 0.130* | 0.119 | 0.053* | 0.327 |
| MyD88 | 0.159 | 0.081 | 0.000 | 0.995 |
| LY96 | 0.426 | ***0.002*** | 0.459 | ***0.001*** |
| IL8 | 0.427 | ***0.002*** | 0.349 | ***0.006*** |
| IRAK2 | 0.423 | ***0.002*** | 0.273 | ***0.018*** |
| HMGB1 | 0.023* | 0.519 | 0.020 | 0.549 |
| SIGIRR | 0.017* | 0.639 | 0.002* | 0.868 |
| TIRAP | 0.002 | 0.847 | 0.004 | 0.790 |

All samples from PTL^+CA^ and PTL^-CA^ were examined. Least squares linear regression (p<0.05) was used. Expression normalised to GapDH. Gene expression assessed by fold change (2^ΔΔCT^).
