# Supplementary material for: Inflammatory Signalling in Fetal Membranes: Increased Expression Levels of TLR 1 in the Presence of Preterm Histological Chorioamnionitis
Source: PLoS One. 2015 May 12;10(5):e0124298. doi: 10.1371/journal.pone.0124298 (PMC4429010; doi:10.1371/journal.pone.0124298)
Supplement: S7 Table — All samples from PTL+CA and PTL-CA were examined. Least squares linear regression (p<0.05) was used. Expression normalised to GapDH. Gene expression assessed by fold change (2ΔΔCT). (DOCX) [file pone.0124298.s007.docx]

S7 Table. The relationship between gene expression and histological staging (fetal).

| **Gene** | **R^2^ (amnion)** | **P value** | **R^2^ (chorion)** | **P value** |
| --- | --- | --- | --- | --- |
| TLR 1 | 0.206 | ***0.044*** | 0.199 | ***0.049*** |
| TLR 2 | 0.397 | ***0.003*** | 0.433 | ***0.002*** |
| TLR 4 | 0.246 | ***0.026*** | 0.008 | 0.714 |
| TLR 6 | 0.068* | 0.266 | 0.067* | 0.271 |
| SARM1 | 0.136 | 0.110 | 0.055* | 0.320 |
| MyD88 | 0.077 | 0.236 | 0.000 | 0.998 |
| LY96 | 0.385 | ***0.003*** | 0.384 | ***0.004*** |
| IL8 | 0.450 | ***0.001*** | 0.502 | ***<0.001*** |
| IRAK2 | 0.489 | ***0.001*** | 0.423 | ***0.002*** |
| HMGB1 | 0.018* | 0.576 | 0.002* | 0.868 |
| SIGIRR | 0.024* | 0.580 | 0.084* | 0.278 |
| TIRAP | 0.064* | 0.283 | 0.048* | 0.355 |

All samples from PTL^+CA^ and PTL^-CA^ were examined. Least squares linear regression (p<0.05) was used. Expression normalised to GapDH. Gene expression assessed by fold change (2^ΔΔCT^).
